# Health impact assessment in two planning projects in England: reflections on normative effectiveness

**DOI:** 10.1186/s12889-024-20203-7

**Published:** 2024-10-14

**Authors:** Thomas B. Fischer, Michael Chang, Tara Muthoora

**Affiliations:** 1 Environment Assessment and Management Research Centre, University of Liverpool, 74 Bedford Street South, Liverpool, L697ZQ UK; 2 Research Unit for Environmental Sciences and Management, Faculty of Natural and Agricultural Sciences, North West University, Potchefstroom, South Africa; 3 Department of Health and Social Care, Office for Health Improvement and Disparities, London, UK

**Keywords:** Health impact assessment, Normative effectiveness, Evaluation, Outcomes, Spatial planning

## Abstract

**Background:**

This article reports on research commissioned by (what was at the time) Public Health England (PHE). The objective is to reflect on the normative effectiveness of Health Impact Assessment (HIA) applied to two planning projects; (a) the new town at Cranbrook, Devon (HIA prepared in 2007), and (b) the regeneration of the Marsh farm area in Luton (HIA prepared in 2009). In this context, the focus is on the contribution of HIA to actions that are intended to lead to good or improved health and wellbeing.

**Methods:**

Normative HIA effectiveness criteria derived from a literature review were used to guide the analysis. The two included HIA cases were previously identified as good practice examples with regards to procedure and report quality. Semi-structured interviews with public health, planning and other actors originally involved in the HIAs were conducted in 2021. This was followed up by web-searches for evidence on actual developments in 2023.

**Results:**

Interviews indicated that normative effectiveness initially appeared to be high, but that the longer-term effects of the financial crash of 2008 reduced this. Delays in initially anticipated timelines and HIA actors moving elsewhere or retiring meant that HIAs were not followed-up and connections between developments and the HIAs were no longer made. However, web-based searches conducted in 2023 found that key HIA suggestions were eventually implemented, albeit with delay. There is also evidence for improved IMD (index of multiple deprivation) rankings in the Marsh farm regeneration case.

**Conclusion:**

A mismatch is observed with regards to HIA exercises appearing to be largely ‘forgotten’ after over a decade of their publication, but recommendations still being implemented, possibly as a result of ‘institutional memory’. Making monitoring and follow-up of HIA binding rather than advisory would allow for direct linkages to be made.

## Key points

- Perceptions of the value of HIA can be strengthened by making it compulsory to follow up recommendations.
- Consideration of HIA monitoring plans should be enforced and go beyond planning consent and onsite works.
- Whilst connections between HIA and development were lost, key recommendations were eventually implemented, even if the HIAs themselves were largely forgotten, hinting at institutional memory surviving over time.
- A wider evidence base should be created on normative effectiveness of HIA.

## Introduction

The use of Health Impact Assessment (HIA) in spatial planning in England has increased substantially over the past years and by 2020 at least around 100 HIAs were conducted annually in this context [1]. Also, in the same year approximately 30% of local authorities mandated supplementary planning documents or local plan guidance to include HIA in certain projects [2]. However, the perception of the value of impact assessments, including HIA, by regulators and political decision makers has tended to be low, in particular with regards to an ability to lead to positive changes on the ground [3]. This paper reports on research commissioned by what was Public Health England (PHE) in 2021 (replaced in 2022 by the UK Health Security Agency and the Office for Health Improvement and Disparities) on how HIA recommendations were translating into implementation practice and, associated with that, improved health and wellbeing.

The ability of HIA to improve health and wellbeing is usually referred to as normative effectiveness [4, 5]. It is not straightforward to establish the extent to which HIA has been normatively effective due to the complexity of causal pathways that are closely associated with long time spans between planning and implementation of projects [6, 20]. A precondition for being able to investigate normative effectiveness is that HIA has been successful in impacting a project plan substantively (i.e. its substantive effectiveness).

Based on previous research for PHE, which graded English spatial plan HIAs for procedural and report quality [1, 18], a database of HIA case studies was created in 2020. From this, a long list of seven high quality HIA cases (as identified by [1]) was considered for further research into normative effectiveness. Two case studies were selected for in-depth analysis and evaluation (NB: resource constraints meant that only two cases could be investigated). On the one hand, this choice was based on long enough timescales between the publication of HIA and the possibility to observe outcomes. On the other hand, it was determined by being able to identify original key actors (local authority representatives and consultants). Considering the time scales, some of those were retired or not detectable (due to e.g. them having moved elsewhere). Furthermore, the implementation status of the associated projects was of importance. Only projects that had reached implementation status could be considered, as otherwise no judgements could be made on normative effectiveness. The two cases meeting those criteria were:

1. Cranbrook New Town Development HIA Main and Technical Report, East Devon (2007) [7, 8] (132 pages; three months preparation time).
2. Marsh Farm Regeneration Programme HIA, Final Report, Luton (2009) [9] (262 pages, nine months preparation time).

## Methodology

The methodology of the research underlying this paper consists of three parts; (1) a literature review for establishing evaluation criteria for normative and substantive HIA effectiveness; (2) interviews with those key actors from the original HIAs that were identifiable with regards to substantive and normative effectiveness criteria; and (3) web-based searches for evidence of normative effectiveness of HIA for the two developments to which they were applied.

The literature review was based on a Scopus® database search for peer-reviewed articles. The search terms "Health Impact Assessment" AND effective*” were used to the title, abstract and keywords of articles, limited to the field of social sciences and English language journal articles between 2005 and mid-2020 (the year the research project was started). This returned 74 articles. Abstracts were reviewed for relevance with regards to actually dealing with the decision support instrument HIA (rather than being on e.g. health monitoring, medical or pharmaceutical interventions), resulting in 30 articles. Only few of these introduced normative and also substantive effectiveness criteria, and the following key criteria were derived for evaluating normative effectiveness, based on [10–14, 14, 15, 19]:

- HIA recommendations have been implemented;
- HIA has contributed to reducing deprivation;
- HIA has contributed to improving health and well-being;
- HIA has contributed to enhanced transparency and scrutiny in project implementation.

Furthermore, the following ‘substantive’ effectiveness criteria were established from the same sources:

- HIA has informed the development making process;
- HIA has led to changes in the project plan;
- HIA has led to the inclusion of specific measures (mitigation or otherwise).

In both HIA cases, evidence was obtained based on interviews. These were conducted with original HIA project staff from the two cases (7 in total) over conference videoing in 2021 (due to Covid19 rules, no physical meetings were possible). Interviewees included:

1. Marsh Farm (Luton)
  1 HIA consultant
  2 Council Public Health Representative
2. Cranbrook (Devon) interviewees
  3; 4: Devon Council Planning Representatives
  5: National Health Service (NHS) Public Health Practitioner
  6: East Devon Council Planning Representative
  7: HIA Consultant

Questions based on the effectiveness criteria were used to guide interviews. Interviews were recorded and transcribed. Subsequently in this paper, citations from these transcripts are used in order to clarify perceptions on normative and also on substantive effectiveness. Finally, in 2023, further evidence was sought through web-based searches on actual developments in the Cranbrook and Marsh Farm areas.

## HIA cases under consideration

Cranbrook was an outline planning application and Masterplan for a new settlement of 2,900 (with potentially up to 8000) homes and a population of up to 20,000, rail and road infrastructure access, social facilities and open spaces in East Devon. The HIA was released in 2007. Construction subsequently started and the first residents moved into their homes in 2012. Development is progressing in several phases up to 2031.

Marsh Farm was an outline planning application for the regeneration of the central area of Marsh Farm Estate (population about 10,000) in Luton. After securing funding from the New Deal for Communities (2001–2010) programme [9], the HIA was commissioned. It was produced prior to planning consent in 2009. By 2021 on-site works of the final construction phase were completed and the community started resettling back.

Both HIAs were prospective and comprehensive in scale. They were standalone and applied next to environmental impact assessments (EIAs). Both HIAs were *opportunistic* in the sense that they were prepared *after* important decisions had been made prior to assessment. This means they were not used to develop options, but rather to assess options that had already been designed (which is typical for current HIA practice; see [16]). However, they were still given space to develop recommendations. Consultants conducted and produced both HIAs. Subsequently, the two cases are introduced in further detail.

### Cranbrook new town development (phase 1, 2007)

‘Private Developers’, the East Devon New Community Partners, submitted a planning proposal which was assessed by East Devon District Council (EDDC) with an HIA to support the development of a 'new vibrant, dynamic and socially cohesive community' ([7], p. 3).

Regional, countywide, and local planning policy for HIA was in place that supported Devon County Council (DCC) in the decision to commission an HIA. This emphasised a need to create a ‘community’ rather than just a ‘physical settlement’ ([7], p. 4). A public health registrar was on the HIA Team, which worked under governance of a Steering Group, composed of planners and public health practitioners. The HIA:

- Assessed how the design of services, transport and connectivity, economy, housing and the built environment, and governance could support social cohesion.
- Took an early decision to scope out aspects covered in the Environmental Impact Assessment (EIA) for the development.
- Developed 'Health Codes' to ‘hard-wire’ health into the strategic principles of the development; these included developer mitigation payments, design requirements, management arrangements to maximise benefits, and monitoring and review, through a series of indicators and metrics of each phase of development ([7], p. 12).

A challenge for HIA public participation was how to profile for a new (i.e. not yet existing) community. This was approached by doing face-to-face and telephone interviews as well as online surveys with stakeholders, including those from neighbouring villages.

HIA recommendations included monitoring on health and deprivation over time, the appointment of a community outreach worker, the construction of a community centre and of a new school which should allow the community to access its facilities. Furthermore, it asked for the widening of childcare options, the reconstruction of the town hall and the establishment of a Town Council. Finally, it made suggestions for ways to establish shared history and identity for the town through arts projects.

#### Marsh farm regeneration programme

This scheme includes the redevelopment of a 1960s council owned housing estate, three miles from Luton town centre with 4,000 dwellings and a population of about 10,000. The design of the original estate had contributed to anti-social behaviour, rising incidences of crime, and increased tension between the police and young people. This led to local riots in 1995 and a legacy of poor community / institutional relations. The regeneration programme, led by the Marsh Farm Community Development Trust (MFCDT) aimed at providing refurbished or new housing, a new supermarket, a pub, gardens and other community facilities with improved pedestrian and vehicular access.

Luton Council had experiences with HIAs from previous planning projects but had no HIA planning policy. The public health practitioner had supportive managers who were said to have understood the benefits of considering health and wellbeing for regeneration projects. The longer-term goal was for the Council to increase its capacity for HIA, and Marsh Farm was seen as a flagship HIA case.

MFCDT assisted the HIA consultant in facilitating equalities-themed workshops with residents from the estate, focusing on children and young people, older people and those with disabilities, ethnic minorities, unemployed people, and tenants of the shopping arcade. They paid participants for their time with shopping vouchers. They adopted this consultation method to bridge the legacy of broken trust between the community and the local authority.

The HIA assigned management for delivery of recommendations to lead agencies. A key aim of the HIA was for the scheme to result in an improvement in the indices of multiple deprivation. In this context, HIA recommendations included the introduction of a new housing policy, with a focus on improved infrastructures next to improved housing. It also provided detailed designs of new open and green spaces, and made suggestions for operational management plans of key services during construction. Relocation strategies worked on the assumption that original tenants were allowed to move back after construction, and suggestions were made for ongoing maintenance and residents’ support plans.

### Indications for substantive and normative effectiveness: Interview results and web-based evidence

Subsequently, we reflect on the normative effectiveness of the HIAs in the light of the results from interviews conducted in 2021 and web-based searches for evidence in 2023. In this context, we first reflect on perceptions of HIA substantive effectiveness.

#### Substantive effectiveness

Overall, perceptions of the HIAs being able to inform and change decisions were positive. In the Marsh Farm case, one interviewee suggested that HIA " *has built in health and wellbeing, so it's an intrinsic part… HIA is good at influencing and shifting mindsets*".

HIAs assessed a range of social determinants of health, with employment, in particular of local people taking centre stage. The Marsh Farm HIA focused on impacts on health inequalities, and "*members of the Community were engaged with thinking about health and about indicators of health, and how that might translate to the community. They talked about things that were troubling them. We also had workshops… the community were very much engaged in it*". On the value of participatory approaches, "*I noticed fairly early on… there's a disconnect from where the Council were, and where the community was in Marsh Farm. We hoped the HIA would bring some of these organisations together and would create a kind of common way forward*".

The Cranbrook HIA found space to consider community cohesion through the provision of social infrastructure. Without an existing settlement, this was a challenge, and "*there wasn't engagement with the developer, we never spoke to the designers…* ". With regards to housing and health, "*they* [initially] *applied a 40% affordable housing target, which meant you had a terrible situation where everyone on the housing lists from both, East Devon and a fair proportion from Exeter were housed in Cranbrook. Without the community infrastructure that you needed to support people… It felt very counter-intuitive trying to make the case* [in the HIA] *for less affordable housing and more community infrastructure*".

Increased scrutiny of the monitoring stages of HIA can bridge a frequently existing evidence gap in providing HIA case studies illustrating health outcomes [7, 14, 19]. However, for Marsh Farm, "*the 25-year monitoring plan didn't happen, I left and there was no one to follow through*". For Cranbrook: "*I look at these health codes* [proposed in the HIA] *and wonder if they had any life beyond the HIA? That would be some interesting learning, if they didn't then why not*?".

In both cases, interviewees stressed how the HIAs had positively influenced receptive institutional leaders. For Cranbrook, "*the HIA was important, fitting into a larger vision of what this development should be*". For Marsh Farm, "*The public health realm in the local authority was increasing, and the authority understood poverty*".

The 2008 global financial crash had an impact on HIA effectiveness in that "*there was a rupture between all the work that had been done. The HIA lost a lot of momentum* " (for Marsh Farm), and "*it made a difference to the power of the developers and what the Council wanted to be done and how much they felt they could… seek to impose health and wellbeing considerations on a market driven solution, as none of the land was publicly owned*" (for Cranbrook).

With regards to an ability to substantively influence development, importantly both cases had enthusiastic champions and leadership for the HIAs and developments. One interviewee suggested that "*Marsh Farm HIA had to be the flagship HIA, in which all the other HIAs base themselves*". The Marsh Farm team involved local community stakeholders, but also built networks within the commissioning organisations. "*It's very rare that you find planners who really can understand and visualise the impact that the plan can have on health and wellbeing, particularly the wider determinants. That's the bit we* [public health] *bring to it*.".

Champions operated within arenas that had experiences with HIA, with individuals that understood HIA well. Luton also had a local HIA policy. Planning and public health actors had different roles, but could decide equally on funding, scope, and methods. The focus on the social determinants of health offered a bridge between planning and public health.

#### Normative effectiveness

Cranbrook HIA recommended the priority scheduling of community infrastructure, which was accepted in the development plan, but which became delayed, in particular due to the 2008 financial crisis. A community outreach worker was employed in 2012, and a new Town Council was established with its first elections held in 2015. In 2019, the District Council submitted a new HIA [17] for Cranbrook's Local Plan extending Phase 1, including decisions on the community centre and other social amenities originally assessed in the 2007 HIA [7, 8].

The construction of a school (Cranbrook education campus) had been completed in 2015 (https://www.cranbrookeducationcampus.org.uk/; last accessed 11/12/2023). The school has allowed for community access, as had been recommended in the HIA. At the time of the interviews in 2021, it was not clear though when exactly community and childcare centre would eventually be delivered. However, this changed in August 2022, when work on Cranbrook Town Centre (including community and childcare centres) started (again, with delay). Importantly, the 40% affordable housing target, which had raised concerns, was not kept. Instead, for the first stage of development, a 30% target was used. Subsequent stages then only applied a 15% target [21]. With regards to HIA recommendations on supporting the arts and cultural activities, a ‘Cranbrook Festival’ initiative was started in 2012, with the aim of bringing ‘together the people of Cranbrook town, Devon, by staging one or more annual arts and community festival events during the year’ (https://cranbrookfestival.com/about-us/; last accessed 11/12/2023). Also, a Cranbrook Cultural Masterplan was prepared in 2015 (https://www.ginkgoprojects.co.uk/cranbrook; last accessed 11/12/2023).

With regards to some initial perceptions of the actual developments once they were under way, one interviewee remarked for the Marsh Farm that initially "*There were some horror stories in the press about these developments, about how they were not working well.*” This was due at least in parts to a delay of the development of social infrastructure, which had been recommended in the HIA. This was eventually implemented though, however only with delay.

On the Marsh Farm Estate, refurbished as well as new housing, a new supermarket, gardens and other community facilities with improved pedestrian and vehicular access were delivered. There is also evidence for ongoing management and support of the local population (for local jobs see e.g. https://marshfarmfutures.co.uk/jobs/; last accessed 11/12/2023). With regards to evidence on how health determinants have developed over time, indices of multiple deprivation (IMD) ranks are considered for the relevant seven lower layer super output (statistical) areas (LSOA), consisting of the following seven domains of deprivation (weighting in brackets; following [22]):

- Income (22.5%)
- Employment (22.5%)
- Health Deprivation and Disability (13.5%)
- Education, Skills Training (13.5%)
- Crime (9.3%)
- Barriers to Housing and Services (9.3%)
- Living Environment (9.3%)

**Fig. 1:**
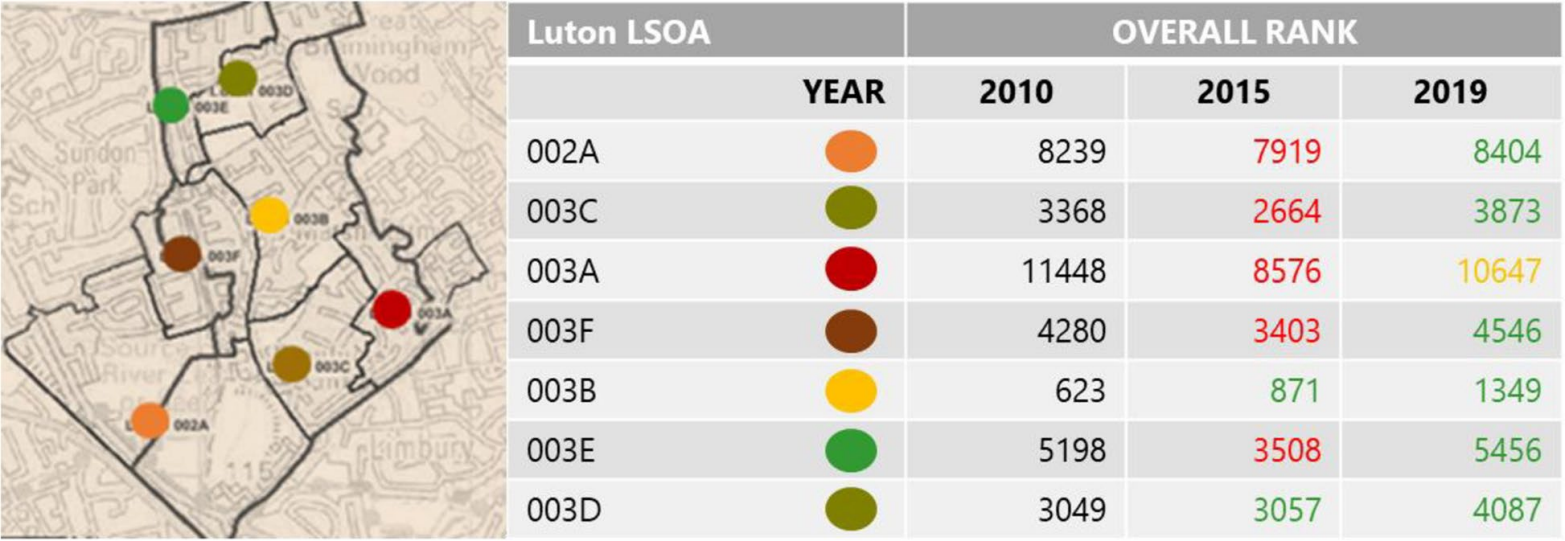
Marsh Farm LSOA (lower layer super output areas) changes in IMD Rank. Source: Adapted from Consumer Data Research Centre *Indices of Multiple Deprivation* (2021)

Neighbourhood level data taken at the time of construction and on completion illustrate that domain rankings had improved between 2015 and 2019, with notable variations (Fig. 1).

The overall rank change worsened during the construction works for all but two of the LSOAs. Following on from that, all but one LSOA had improved in rank from 2010 at the start of the development to 2019 at completion and resettlement of the 2nd construction phase (with the 3rd and final construction phase being underway).

Table 1 summarises results discussed above with regards to normative effectiveness for key HIA recommendations. This indicates that the HIAs were normatively effective to some extent, the two exceptions being serious shortcomings in monitoring and delays in delivering support infrastructures.

**Table 1:** Normative effectiveness-implementation of some key HIA recommendations

***Cranbrook development***	✓ = met ✘ = not met ? = unclear
Monitoring on health and deprivation over time	✘
Priority scheduling of community infrastructure	✘
Appointment of a community outreach worker	✓
Construction of a community centre	✓
Construction of a new school which allows the community to access its facilities	✓
Widening of childcare options,	✓
Reconstruction of the town hall	✓
Establishment of a Town Council	✓
Reducing sustainable housing target to less than 40%	✓
Conducting arts projects	✓
***Marsh farm development***	
Improving indices of multiple deprivation	✓
Improving infrastructures at the same time of new housing	✘
Detailed designs of new open and green spaces; gardens, improved access	✓
Relocation strategies; allowing original tenants to move back after construction	✓
Operational management plans of key services during construction	?
Ongoing maintenance and management plans	✓

## Limitations

Observing changes in health and wellbeing outcomes as a consequence of spatial planning takes time. Even though it had been over a decade since the assessments were conducted, it was arguably still early to assess normative outcomes. However, over a decade is a considerable part of a personal career, and the aim was to interview original HIA personnel, who were still working in public health and planning. This was a challenge because e.g. personnel had left, there were no contact details, and there was wariness of being ‘evaluated’. A weakness of IA research is the role of the private sector; where sharing of knowledge is monetarised, findings are business sensitive and expectations in editing approvals need managing [13, 23]. To help counter these limitations, PHE emphasised that the research was not an evaluation but an examination of effectiveness of HIA in spatial planning. Finally, it is not possible to clearly connect observed effects with any particular document, as numerous other planning documents and strategies are usually prepared that go hand in hand with the HIAs.

## Conclusions

The objective of this paper was to examine normative effectiveness of HIA in English spatial planning on the basis of two case studies that had previously been identified as examples of good practice with regards to how they were conducted procedurally and the quality of documentation; Cranbrook New Town in Devon and Marsh Farm Estate in Luton. The contexts of the case studies were supportive in terms of individual actors, host communities, institutional operations, approval from leadership, or by policy/funding objectives. Contextual support changed in response to external factors, namely the 2008 financial crash, stalling long-term HIA monitoring. Whilst the scale of development means that in parts it was still early to assess normative effectiveness in 2021 to 2023, we observed some promising developments. In this context, neighbourhood indices for regeneration outcomes in Marsh Farm were particularly encouraging. This was associated with key HIA recommendations being implemented, albeit with some delay. For the Cranbrook case, whilst HIA recommendations such as the employment of a community outreach worker had been taken up early on, budgetary constraints following the 2008 financial crash meant that this didn’t initially continue. Evidence from 2023, though, shows that all key HIA recommendations had eventually found their way into practice (including construction of a community centre and a new school which allowed the community to access its facilities).

Directly linking the HIAs with observed outcomes was somewhat difficult, in particular as HIA monitoring was deficient. This means that whilst HIAs have not been able to contribute to enhanced transparency and scrutiny in project implementation, key recommendations survived, possibly due to institutional memory and also because HIA recommendations reflected what communities wanted. With regards to lack of monitoring, when applied next to EIA, HIA monitoring should be integrated with EIA monitoring. If applied on its own, HIA monitoring should become statutory.

Finally, it is important to acknowledge that HIA has a weak policy status, and efforts are needed to create a stronger enabling context (e.g. HIA recommendations being binding rather than advisory). Importantly, further systematic research on HIA follow-up is urgently required, in particular in the light of some negative perceptions by decision makers.

## Data Availability

The datasets used and/or analysed during the current study available from the corresponding author on reasonable request.
